# Alternative Splicing in Heat Shock Protein Transcripts as a Mechanism of Cell Adaptation in *Trichophyton rubrum*

**DOI:** 10.3390/cells8101206

**Published:** 2019-10-05

**Authors:** João Neves-da-Rocha, Tamires A. Bitencourt, Vanderci M. de Oliveira, Pablo R. Sanches, Antonio Rossi, Nilce M. Martinez-Rossi

**Affiliations:** Department of Genetics, Ribeirão Preto Medical School, University of São Paulo, Ribeirão Preto-SP 14049-900, Brazil; tabitencourt@gmail.com (T.A.B.); cuca@fmrp.usp.br (V.M.d.O.); psanches@usp.br (P.R.S.)

**Keywords:** *Trichophyton rubrum*, dermatophytes, heat shock proteins, HSPs, alternative splicing, cell adaptation, stress response

## Abstract

Heat shock proteins (HSPs) are involved in critical processes like host tissue invasion, resistance, and pathogenicity in dermatophytes. RNA-Seq analysis of *Trichophyton rubrum* exposed to undecanoic acid (UDA) revealed intron retention events in HSP transcripts. Because HSPs are modulated in response to various stimuli and as alternative splicing (AS) can result in a broad diversity in the proteome of eukaryotic cells, our objective was to confirm the aforementioned retention events, investigating their consequences and extent. Furthermore, we aimed to determine: (1) the expression profile of HSP genes in an infection-like scenario and (2) the importance of Hsp90 for the keratinolytic potential of *T. rubrum*. RT and qPCR analyses comparing the exposure to UDA and terbinafine (TRB) confirmed the presence of two mRNA isoforms of the *hsp7-like* gene, with distinct expression patterns in response to UDA and TRB. The HSP expression profile revealed two upregulated, three downregulated, and four unmodulated transcripts; Hsp90 inhibition by 17-AAG resulted in a significant decrease in keratinolytic potential at 37 °C. Altogether, these results broaden the current knowledge on the importance of HSP-mediated pathways for cell adaptation and other aspects of dermatophyte biology, indicating that HSP network proteins can be potential targets for antifungal therapy.

## 1. Introduction

Heat shock proteins (HSPs) are a highly conserved group of chaperones found in virtually all living organisms, both prokaryotes and eukaryotes [1,2]. Sharing the characteristic attribute of having their expression rapidly modulated by various stress conditions, HSPs act at different stages to regulate the following features of target proteins: folding, maintenance of native conformation, and fragmentation of aggregates. Simultaneously, they are also involved in transport and degradation pathways, signaling, and cell cycle regulation [2,3,4]. In regards to fungi, a robust literature can be found relating HSPs to the regulation of the life cycle of these organisms or an increase in survival rates under stress conditions [5,6]. In dermatophytes, the presence of these chaperone proteins is also fundamental for several aspects of their biology: from the early stages of invasion into the host organism to their degree of pathogenicity and resistance to antifungal drugs [7,8].

Anthropophilic dermatophytes are responsible for causing chronic, slow-progression infections in humans as a result of long-term co-evolutionary dynamics with hosts. Keratinized body parts such as hair, skin, and nails are those most affected, with colonization usually remaining limited to the outermost layers of tissues; nonetheless, such infections may assume a severe and invasive character in immunocompromised patients [9,10,11,12]. Several studies have been developed to identify the prevalence of certain dermatophyte species in correlation with their forms of infection and specific regions of the planet [13,14,15,16,17]. *Trichophyton rubrum*, in particular, appears in a prominent position in these studies, being in some cases highlighted as the main species causing dermatophytosis worldwide [16,17].

Physicochemical changes in the extracellular medium may be responsible for determining a wide metabolic reprogramming in fungal cells, a process which is known to involve the control of gene expression through the processing of primary transcripts [18,19,20]. A recent study in dermatophytes demonstrated that spliceosome factor genes can be transcriptionally modulated by a plethora of stimuli, including antifungal agents [21]. Therefore, the importance of alternative splicing (AS) for the adaptation of fungi has been progressively reinforced. In this context, we must consider the associations previously established [22,23], which highlight the occurrence of AS by intron retention in *Ascomycota*, with greater prevalence in filamentous, pathogenic species. Additionally, recent studies demonstrate the importance of investigating such post-transcriptional regulatory mechanisms, also correlating them with adaptive acquisitions and fungi pathogenicity [24,25,26].

Post-transcriptional regulation by AS is a process which enables the generation of different versions of a given protein (isoforms) according to physiological needs and environmental stimuli, often representing a primary source of phenotypic diversity within the proteome of eukaryotic cells [27,28,29]. Therefore, the modulation and occurrence of AS in HSP-encoding transcripts might provide dermatophytes with adaptive advantages in response to various stimuli. The objective of this study is to investigate the occurrence of intron retention events in the transcripts of Hsp70 family members, and to discuss the role played by the regulation of HSP-mediated networks in cell adaptation in *T. rubrum*. We elucidate some of the details concerning the strategies employed by dermatophytes in response to antifungal therapy and changes in the extracellular environment during the colonization of keratinized tissues.

## 2. Materials and Methods

### 2.1. Strains and Culture Conditions

*T. rubrum* strain CBS 118892, obtained from the Centraalbureau voor Schimmelcultures, Fungal Biodiversity Centre (Utrecht, Netherlands), was cultured on malt extract agar solid medium (MEA: 2% glucose, 2% malt extract, 0.1% peptone, 2% agar, pH 5.7) at 28 °C for 20 days [30]. To prepare conidia suspensions, plates were flooded with sterile 0.9% NaCl solution, and the suspension was filtered through fiber glass. The conidia concentration was then estimated using a Neubauer chamber.

### 2.2. Alternative Splicing Assay in Response to Drugs

For the alternative splicing assay, approximately 1 × 10^6^ conidia were inoculated into 100 mL of Sabouraud medium and cultured at 28 °C for 96 h under constant agitation (120 rpm). For sampling time points at 3 and 12 h, the mycelia were filtered and incubated in the presence or absence of 70% of the minimum inhibitory concentration (MIC) of undecanoic acid (UDA; 35.0 μg/mL) or terbinafine (TRB; 0.014 μg/mL) in Sabouraud medium. Mycelia were subsequently stored at −80 °C until RNA extraction. The assay was carried out in triplicate.

### 2.3. Co-Culture Assay with Human Keratinocytes

The co-culture assay was carried out as described previously [31], with modifications. A conidia solution (2 × 10^6^ conidia/mL) prepared in RPMI 1640 medium containing 5% serum was inoculated into a culture of immortalized human keratinocytes (HaCaT; 2.5 × 10^5^ cells/mL). The culture was then incubated at 37 °C in a greenhouse containing 5% CO_2_ for 24 h. The assay was carried out in triplicate.

### 2.4. Keratinolytic Assay

For the keratinolytic activity assay, approximately 5 × 10^5^ conidia were inoculated in 25 mL of water + keratin medium (2.5 g/L) in the presence or absence of the Hsp90 inhibitor 17-AAG (17-allylamino-17-demethoxygeldanamycin; InvivoGen, San Diego, CA, USA), at a concentration of 14.65 μg/mL (25 μM). Culture was performed at pH 5.0 and was maintained at 37 °C or 28 °C for seven days under constant agitation (120 rpm). After the incubation period, the cultures were filtered, and supernatants and mycelia were recovered. The evaluation of keratinolytic potential was performed according to a previously published protocol [32], with modifications. Keratin was used as the substrate and 1.0 mL of culture medium as the enzyme. The test was carried out at pH 8.0. Mycelium-specific activities were expressed as units per gram of mycelium dry weight. The assay was carried out in triplicate.

### 2.5. RNA Extraction and cDNA Synthesis

Total RNA extraction from *T. rubrum* mycelia was performed using the Trizol reagent (Thermo Fisher Scientific, Carlsbad, CA, USA), according to manufacturer instructions. Samples were treated with DNaseI (Sigma-Aldrich, St. Louis, MO, USA) to eliminate all DNA traces, and cDNA synthesis was carried out using the High-Capacity cDNA Reverse Transcription Kit (Applied Biosystems, Foster City, CA, USA), following the manufacturer’s recommendations. RNA and cDNA concentrations and quality were assessed using Nanodrop (Thermo Fisher Scientific) and also by performing a reverse transcription polymerase chain reaction (RT-PCR) for β-tubulin, a constitutive gene. Total RNA from co-culture assays was extracted using the Illustra RNAspin Mini RNA Isolation Kit (GE Healthcare, Little Chalfont, UK), as previously described [33].

### 2.6. In Silico Characterization of HSP Isoforms

Intron retention events involving HSP-encoding genes were identified from the RNA-Seq data of *T. rubrum* exposed to UDA [34]. The genes and isoforms identified were analysed for their sequences, reading frames, and conserved sites and domains using in silico analysis tools and databases such as Ensembl Fungi (https://fungi.ensembl.org/index.html), ExPASy Translate Tool (https://web.expasy.org/translate/), Interpro [35], and Pfam [36]. The structural representation of isoforms was designed using the Illustrator for Biological Sequences software (IBS) [37].

### 2.7. Gene Expression Analysis

Oligonucleotide sequences (Table 1) were designed using the IDT DNA primer quest tool (http://www.idtdna.com/primerquest/Home/Index) and analyzed using Oligoanalyzer 3.1 (https://www.idtdna.com/calc/analyzer), and Blast (http://blast.ncbi.nlm.nih.gov/Blast.cgi). For the AS study, confirmation of the presence of isoforms was performed by RT-PCR prior to quantitative analysis; in this case, primers flanking the intron of interest were used. As for quantitative polymerase chain reactions (qPCR), two primer pairs were designed for each gene, one of them was located inside the intron of interest, and allowed the evaluation of the presence of the retained isoform, and the other was present in an exon region, and enabled the quantification of total gene expression.

For qPCR reactions of all expression assays, standard curves were generated for each primer pair using a DNA sample at five-point, two-fold dilutions and measured in duplicates. Oligonucleotides had their concentration standardized for reaction efficiencies between 90% and 110%. The reactions were prepared using Sybr Green and PCR Master Mix (Life Technologies, Carlsbad, CA, USA) under conditions recommended by the manufacturer, and the ROX dye was used as the fluorescence signal normalizer. The platform used was QuantStudio v. 3 ( Thermo Fisher Scientific) and the fold change was calculated using the relative quantification ΔΔ Ct [38]. All reactions were performed in triplicate in 96-well plates using the reference genes *rpb2* and *gapdh* for data normalization, as previously described [39].

Statistical significance was evaluated by one-way ANOVA followed by the Tukey’s ad hoc test, or by the unpaired t-test alone. The software used for both statistical analyses and graphs was the GraphPad Prism v. 5.1 (GraphPad Software, San Diego, CA, USA).

## 3. Results

### 3.1. Alternative Splicing Assay

**Figure 1:**
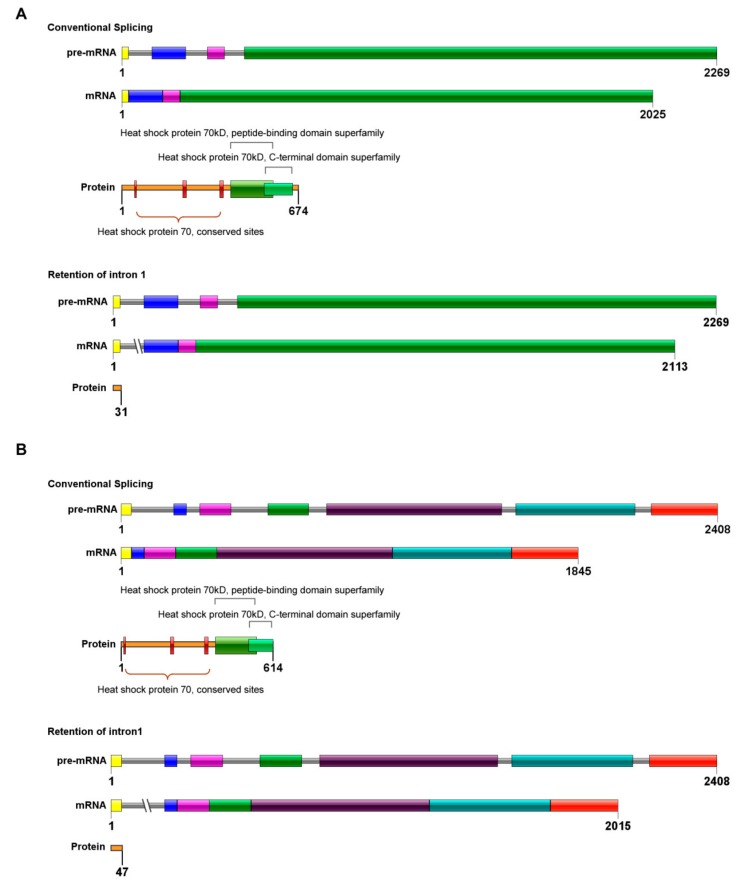
Schematic representation of Intron1 retention in Hsp70 family members in *Trichophyton rubrum*, showing DNA organization, mRNA, and proteins resulting from conventional splicing and alternative splicing promoted by Intron1 retention. Exons are shown as colored boxes; introns are shown as solid lines. Premature stop codons are indicated by cut lines. Conserved sites and domains are indicated in the structure of the proteins. (**A**) *hsp7-like* gene. (**B**) *hsp75-like* gene.

The RNA-Seq analysis of *T. rubrum* exposed to the antifungal agent UDA [34] suggests the occurrence of intron retention events in two of the fungus HSPs-encoding genes: *hsp7-like protein* (TERG_03206) and *hsp75-like protein* (TERG_01883) (Supplementary Table S1). In silico investigations performed for both genes demonstrated that conventional processing of their respective pre-mRNAs produces functional proteins, both belonging to the Hsp70 family (Figure 1). Moreover, AS events by intron retention promote disruptions in the open reading frames (ORFs) that result in mRNAs with premature stop codons, possibly associated with the generation of truncated or non-functional proteins (Figure 1).

To validate the indications provided by transcriptome analyses, the aforementioned splicing events under UDA exposure were investigated by RT-PCR, with subsequent evaluation by qPCR for quantitative accuracy. Moreover, searching to broaden our understanding on the extent of post-transcriptional regulation by alternative splicing in these HSP genes, we determined the expression profile of retention events under exposure of *T. rubrum* to terbinafine (TRB), another antifungal drug of medical use, in this case, affecting the synthesis of ergosterol by inhibiting squalene epoxidase. Thus, it becomes possible to draw a comparison between the regulatory profiles of the transcripts in response to two different stimuli.

**Figure 2:**
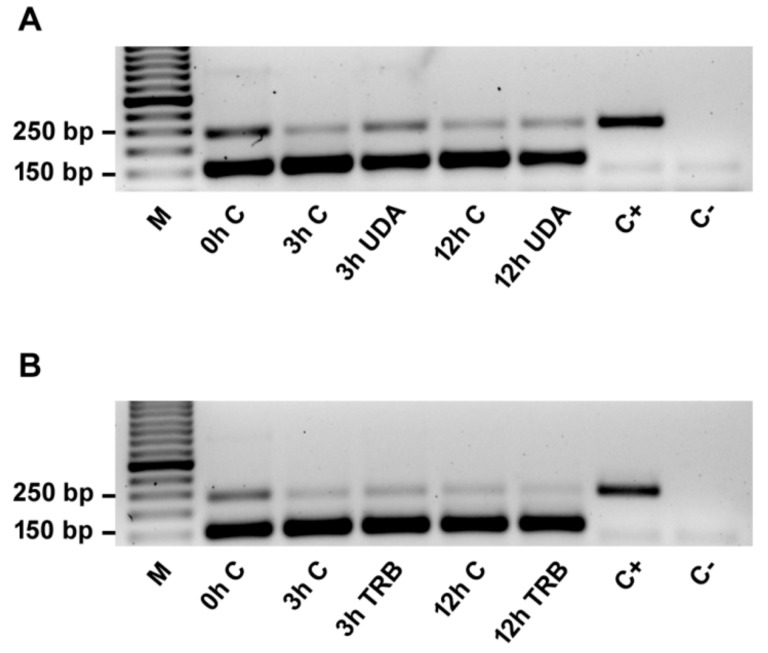
Intron1 retention after pre-mRNA processing of the *hsp7-like* gene visualized by electrophoresis following RT-PCR. *T. rubrum* was exposed to undecanoic acid (UDA) (**A**) and terbinafine (TRB) (**B**) for 0 h (control), 3 h, and 12 h. (M) Molecular weight ladder; (C+) positive control; (C−) negative control. The size of each amplicon was 153 bp for the processing of Intron1 and 241 bp for the retention of Intron1. The full-length gel is shown in Supplementary Figure S1.

**Figure 3:**
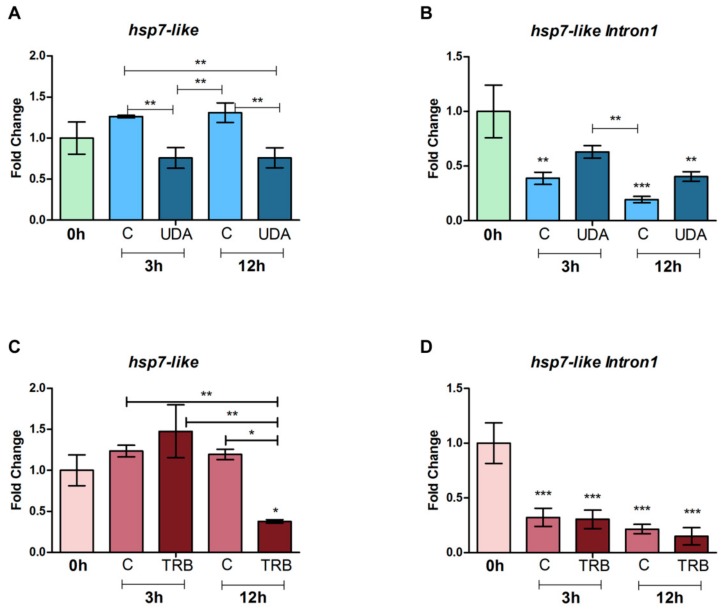
qPCR results for the *hsp7-like* gene after growth in the presence or absence of the antifungal drugs UDA and TRB for time points 0 h, 3 h, and 12 h. Expression levels are represented in comparison to the 0 h control. (**A**) Total expression, UDA exposure. (**B**) Intron-1 retention expression, UDA exposure. (**C**) Total expression, TRB exposure. (**D**) Intron-1 retention expression, TRB exposure. Data are means ± Standard deviation (SD). (**C**) Paired control. Significant statistical differences are represented by asterisks (* *p* < 0.5, ** *p* < 0.01, *** *p* < 0.001).

The RT-PCR results indicate the occurrence of both isoforms of *hsp7-like* transcripts under the conditions tested, with retention events detected with stronger bands in 0 h control (Figure 2). In general, the occurrence of the intron-retained isoform was verified in all experimental conditions and biological replicates, with subtle differences in band intensity. The results from qPCR reveal that in the case of UDA exposure, the overall level of gene expression was maintained, regardless of the time (comparison of 0 h, 3 h control, and 12 h control samples among themselves, and 3 h UDA versus 12 h UDA) (Figure 3A). Despite the relative increase in transcript levels observed at 3 and 12 h under control conditions, compared to the starting point (0 h), this was not statistically significant. As for the factor presence/absence of the drug, a decrease in total expression was observed under UDA if contrasted to paired controls, with statistical support for both 3 h and 12 h time-point samplings. With respect to the retained isoform, a general decrease in expression was observed for other conditions relative to the 0 h control (Figure 3B). The difference between 3 h UDA or 12 h UDA samples and their respective paired controls did not obtain optimal statistical support; nonetheless, a pattern of upregulation of the retained isoform in response to UDA was strongly suggested (Figure 3B).

For the TRB-exposure data, the qPCR results reveal quite a distinct pattern, in which maintenance in the total level of expression of the gene over time only held for control conditions (Figure 3C). On comparing 12 h TRB with 3h TRB and 12 h control, a radical decrease in expression was observed; therefore, this overview can be considered informative for the two factors investigated here, incubation time and drug presence, both with robust statistical support. Interestingly, the regulation of Intron1 retention events was initiated at the first time-point samplings, with significant downregulation being observed from 3 h, while expression levels remained highly stable among all paired exposure times (Figure 3D).

The retention events detected in RNA-Seq data for *hsp75-like* transcripts were not validated by our experiments (data not shown). This fact suggests that the values that are presented in Supplementary Table S1 for this gene are somehow overestimated. Further in silico investigations have already been carried out in order to carefully examine this issue and confirmed a lack of consistency in the transcriptome analysis for this specific gene. Notwithstanding, since the same regulatory mechanism concerning the first intron would also be possible for this gene (see Discussion), we suspect that the regulation of its transcripts by AS might well take place under certain conditions.

### 3.2. HSPs Expression Profile in An Infection-Like Scenario

After validating the RNA-Seq data regarding the events of intron retention in the *hsp7-like* gene and establishing a perspective with respect to the regulation of this post-transcriptional processing under two different cellular stimuli related to medical antifungal therapy, our main goal was to determine the expression profile of representative HSP genes in the genome of the fungus (*hsp30*, *hsp60*, *hsp7-like*, *hsp75-like*, *hsp88-like*, *hsp90,* and two *hsp104* genes) in an in vitro, infection-like scenario. Towards this, *T. rubrum* co-cultured with the human keratinocyte cell line HaCaT was used; this model is considered to be analogous to a dermatophyte skin infection.

A survey of the genes of interest revealed some inconsistencies in the descriptions of HSPs in the Ensembl Fungi database when taking into account old annotations of the same genes and also their molecular characterization in comparison to the reference genes in the genome of *Saccharomyces cerevisiae*. We characterized the sequences identified as TERG_07949 and TERG_12507 in *T. rubrum* as paralogs and orthologous to the *Hsp104* gene in *S. cerevisiae* (YLL026W), in silico; moreover, TERG_07658, which commonly appears in publications as *hsp88-like*, but is found in the Ensembl database as a hypothetical protein, had its structure predicted and redefined here for the purposes of identifying the actual coding sequence. In fact, we found that this gene actually belongs to the Hsp70 family. The analyses that led us to such conclusions can be found in the Supplementary Materials (Figures S2 and S3).

**Figure 4:**
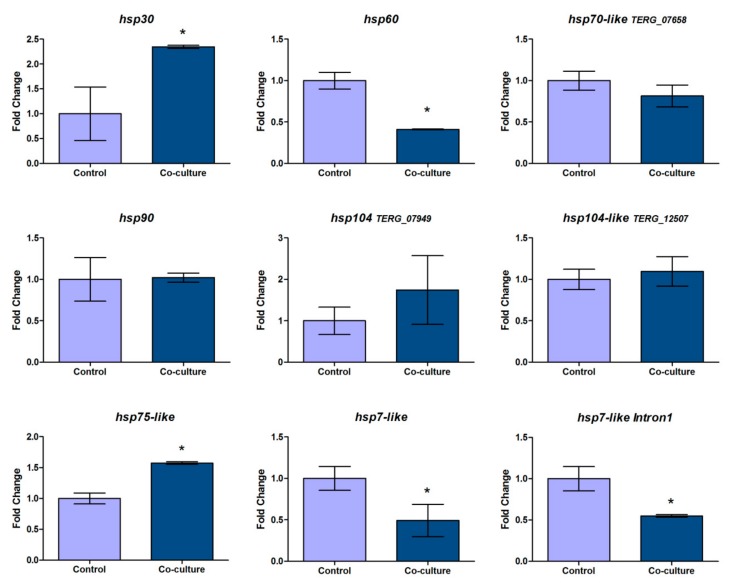
qPCR results for the HSP expression profile of *T. rubrum* after co-culture with human keratinocytes. Expression levels for each condition are represented in comparison to the control (*T. rubrum* grown alone). Data are means ± SD. Significant statistical differences are represented by asterisks (* *p* < 0.5).

The results for the selected genes revealed an expression profile of HSP comprising two upregulated transcripts (*hsp30* and *hsp75-like*), three downregulated (*hsp60*, *hsp7-like,* and the *hsp7-like Intron1*), and four not significantly modulated transcripts (*hsp90*, *hsp70-like,* and both of the *hsp104* genes) under the infection-like scenario investigated in this study (Figure 4).

### 3.3. Evaluation of the Keratinolytic Potential of T. rubrum under Hsp90 Inhibition

Following our investigation on the role of HSPs in the pathogenicity and adaptability potential of *T. rubrum* under infection-like and stressful circumstances, we sought to examine whether the depletion of Hsp90 cellular levels (as should occur under various environmental stress conditions) might be involved in modulating the activity of keratinolytic proteases in *T. rubrum*, through molecular inhibition tests.

Hsp90 is a central component of HSP-mediated pathways and directly associated with the chaperoning of diverse proteins, including signal transducers [3,40]. Under normal conditions, it has a naturally excessive, pre-adaptive, and buffering presence in cells; only under stress conditions is Hsp90 more recruited and its excess ultimately consumed [41]. Despite the fact that no modulation in *hsp90* transcript levels was detected in the co-culture assay (Figure 4), previous studies strongly suggest the importance of this chaperone for *T. rubrum* infection in keratinized body parts [7], which might be related to the ability of the fungus to degrade keratin.

**Figure 5:**
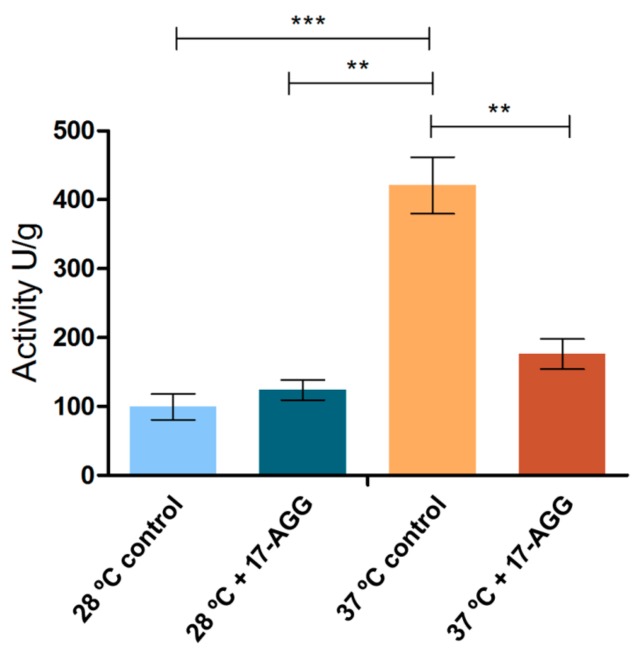
Evaluation of *T. rubrum* keratinolytic potential after growth in the presence or absence of the *hsp90* inhibitor 17-AAG at 28 °C or 37 °C. Keratinolytic activity is calculated in unities/g (U/g). Data are means ± SD. Significant statistical differences are represented by asterisks (** *p* < 0.01, *** *p* < 0.001).

A keratinolytic assay was conducted and revealed a significant decrease in activity when the fungus was cultured in keratin supplemented with 25 μM of Hsp90 inhibitor 17-AAG at 37 °C, compared to the water + keratin control at the same temperature. No significant difference was observed at 28 °C (Figure 5).

## 4. Discussion

Alternative splicing is a post-transcriptional regulatory mechanism that might determine a broad phenotypic diversity in the proteome of eukaryotic cells, according to environmental conditions. The presence of HSPs in the genome of *T. rubrum* and other dermatophytes has been shown as fundamental for several aspects of their biology [8]. Our investigations on AS and modulation of HSP transcripts unraveled essential issues regarding the role of such proteins in cell adaptation in *T. rubrum*.

The fact that RNA-Seq analysis in a previous study [34] found retention events of the first intron of HSP transcripts with occurrence mostly restricted to the 0 h time-point sampling under control conditions (Supplementary Table S1) could indicate a regulation towards conventional processing under cellular stress provoked by the presence of UDA, with all introns being spliced out during mRNA maturation. From this perspective, the formation of truncated mRNAs would be more common in the absence of the drug (i.e., steady, unstressed cellular conditions), presumably due to a lack of stimuli in favor of the usage and recruitment of HSPs. However, despite confirming the referred transcriptome findings, our data showed that when paired 3 and 12 h controls are taken into account, along with total gene expression, this pattern is not sustained, as Intron1 retention levels were shown to increase in the presence of UDA, while total expression significantly decreased (Figure 3A,B). Thus, the proposition we advocate for interpreting these data is that of a relative decrease in the amount of transcripts coding for functional proteins under UDA exposure as a result of both an effect of time of growth and development of *T. rubrum*, and to either a deregulation in the processing of *hsp7-like* transcripts or an inhibition of this gene in some of the pathways in which it is involved.

UDA is a medium length chain fatty-acid with non-specific action on fungal cells; TRB, in turn, belongs to the class of allylamines, with effects on the synthetic route of ergosterol [9]. In response to this drug, data suggest that regulation of *hsp7-like* total expression only takes place at the latter times of exposure (Figure 3C). No modulation in Intron1 retention was detected in response to TRB (Figure 3D); nonetheless, retention events were still highly correlated with fungal development, with a general increase in the amount of molecules coding for functional proteins from 3 h conditions.

Comparison of the UDA and TRB data supports the existence of a regulatory pathway for the processing of *hsp7-like* transcripts, presumably resulting in the optimization of the HSP-mediated response according to cellular conditions. By interfering with the structure of ORFs, the retention events described here would certainly prevent unnecessary energy expense during translation. It seems reasonable that this mechanism should rely upon the first intron of genes: for the circumstances described (Figure 1), we suspect that translation may not even be initiated. Refined mechanisms for dynamic optimization are standard features in eukaryotic cells and have been described recently for different processes related to protein production and metabolism [42,43,44,45,46,47].

Hsp70 family members integrate different HSP pathways in a highly centralized and coordinated manner. In summary, our results indicate that retention of Intron1 in specific ratios during pre-mRNA splicing acts as a regulatory mechanism for *hsp7-like* transcripts in *T. rubrum*, which might be related to cell adaptation. We advocate that such regulation might occur as a compensatory mechanism associated with controlling the turnover of RNAs, which include Hsp70 family transcripts. In this chaperone family, paralogs respond differently to specific conditions (see later in the Section 4) and their genetic arrangement typically enables Intron1 retention interference with the structure of ORFs, as described (data only shown for TERG_01883 and TERG_03206, Figure 1).

Validation of intron retention events for the same transcriptome data was performed for the *impdh* and *pakA/ste20* genes [34,48]. For the *impdh* gene, results show a decrease in the rates of Intron2 retention in the presence of UDA at 12 h, compared to 3 h; such events also result in proteins with interrupted functional domains [34]. As for *pakA/ste20* protein kinase, a pattern of Intron1 retention resulting in premature stop codons and prevalent under UDA exposure was highlighted at 3 h [48]. Such genes have functions that largely differ from those performed by HSPs; for this reason, the fact that retention patterns observed in these cases are different to the one verified herein for *hsp7-like* transcripts indicate the ability of *T. rubrum* to coordinate AS events. We believe that our research on the processing of HSPs transcripts provided reliable and detailed information on the occurrence of AS in *T. rubrum* in response to drugs, as it takes into consideration all paired control conditions, overall gene expression profile, and the exposure to different antifungal agents.

The regulation of heat shock proteins is a critical factor for promoting a quick and efficient response to several stressful conditions. Among the stimuli capable of triggering HSPs, we highlight in dermatophytes those related to the infection of keratinized tissues [7,49,50], a process which promotes profound biochemical modifications in the surrounding medium [10,11,50]. One of the determining factors for the success of these organisms in colonizing host tissues is their ability to adjust their metabolism rapidly according to chemical and nutritional variations, a factor intimately associated with the presence of keratinolytic proteases and efficient response pathways to environmental stimuli [10,50].

The in vitro model used here to simulate a skin infection by *T. rubrum* unraveled a significant modulation of the transcripts of HSP genes from different families. This approach allowed us to establish a novel overview of HSP modulation in the context of interaction with living cells, identifying potentially important genes for the *T. rubrum* infection process. Furthermore, molecular inhibition of the Hsp90 protein, a central component of HSP-mediated pathways, revealed a substantial decrease in the activity of *T. rubrum* keratinolytic proteases at 37 °C, the approximate temperature of the human body. These results strengthen the view of such chaperones as essential housekeeping components not only for the maintenance of basic cell functions, but additionally as entities integrating the metabolic adaptations and specificities of organisms through network rewiring over evolution [40]. Since Hsp90 is intimately associated with transistor functions, tuning physiological responses to environmental conditions by interacting with signal transducers [3,40], it is likely that its inhibition might affect the ability of the cell to respond to extracellular stimuli in general, resulting in the observed decrease in keratinolytic activity and also in a reduced capacity to grow on nails, as well as an increased susceptibility to antifungal compounds [7].

Altogether, we consider that the proteins belonging to the Hsp30, Hsp60, Hsp70, and Hsp90 families appear to play some role during *T. rubrum* growth in keratinized tissues. We also confirmed the occurrence of splicing events in *hsp7-like* transcripts for the infection-like scenario: both total gene expression and intron retention levels were downregulated (Figure 4). Most interestingly, we noticed that Hsp70 family members exhibited completely different expression patterns in the co-culture assay, corroborating the notion already implied in previous studies [7] that, despite their identity, these proteins might have differentially adapted as paralogs to specific stimuli in the course of evolution. Thus, we suggest that possible intron retention events in other transcripts of the Hsp70 family may occur in different patterns and with specific responses for each of these paralogs. This hypothesis should favor the fine regulation of pathways during fungal development, response to environmental stimuli, host–pathogen interaction, and so on.

Furthermore, we sustain that the apparent contrast concerning Hsp90 modulation in the previous assays should by no means be considered an incongruity: such results can be easily explained by (1) the naturally excessive, buffering presence of Hsp90 in the proteome of cells [41]; (2) a compensatory regulation in HSPs pathways during infection; and lastly, although equally important, (3) a mere indirect influence of the depletion in Hsp90 levels on *T. rubrum* keratinolytic potential, with no direct modulation at transcriptional level during infection. These hypotheses are not considered mutually exclusive.

The effects of Hsp90 inhibition, observed due to diminished keratinolytic potential, corroborate numerous indications of the importance of this chaperone in the *T. rubrum* infection process. In fact, our findings substantiate and complement those of previous studies [7], in which in vitro and ex vivo assays were carried out to evaluate the expression of HSPs and the role of Hsp90 under different scenarios. In this context, infection models and Hsp90 inhibition reveal informative modulation patterns for many genes (including those investigated here), which, in turn, correlate with the general overview of HSPs in dermatophytes [8]. It is worth noting that the annotation of these genes has largely changed over the years due to database updates; nonetheless, accession numbers have mostly remained the same in all studies concerning HSPs in *T. rubrum*.

These results do not fully confirm the formulated hypotheses, which require further testing. In addition, the patterns observed and described for HSP regulation might vary for other infection models and *T. rubrum* strains. In a real infection scenario, several factors, such as skin pH and temperature, inflammatory response, and oxidative stress, act together to indirectly modulate the response by HSPs. Nonetheless, we believe that our results broadly support the role of HSPs in cell adaptation in *T. rubrum* at many regulatory levels.

**Figure 6:**
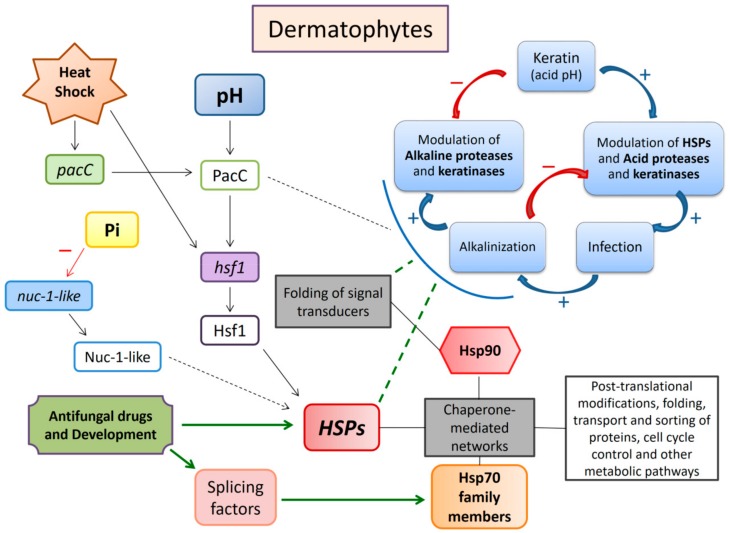
An extended model of HSP regulation and networking in dermatophytes in response to extracellular stimuli, such as heat shock, pH variations, Pi levels, keratin, and drugs. Broken lines correspond to metabolic pathways that have not yet been fully elucidated. Stronger, green lines and arrows indicate those pathways to which our contributions are thought to be relevant. We point out that the factor identified here as Nuc-1-like is only a reference to that of *Neurospora crassa*, since genes involved in phosphate metabolism are not yet well studied in dermatophytes. In order to assemble and summarize the most relevant data on the subject, we undertook a wide literature survey and used as reference the models already proposed [8,11].

We tried to assemble and correlate most of the available data regarding HSPs in dermatophytes in the scheme presented in Figure 6. In summary, we sustain that HSPs play an important role during the fungal infection of keratinized tissues, mainly through the effects of Hsp90 monitoring on signal transduction, which regulates cell responses to environmental stimuli. Based on our results, we also suggest that, at least for some species, AS must relate to the regulation of Hsp70 family members, being associated to both fungal development and drug presence or other stress conditions. Furthermore, the modulation of gene expression levels by Hsf1, PacC, and Nuc-1-like transcriptional regulators is thought to be essential for the fine tuning of HSP-mediated response mechanisms that dermatophytes possess for cell adaptation.

## 5. Conclusions

Our findings highlight Hsp70 family members as relevant candidates to undergo post-transcriptional regulation by AS during development and in response to extracellular stimuli in *T. rubrum*. Furthermore, we suggest that the compensatory mechanisms already described for such chaperone proteins may now also be said to be post-transcriptionally regulated by splicing factors, not simply representing a modulation by heat-shock transcription factors (HSFs) at DNA level. To our knowledge, this is the first time that the regulation of HSPs by AS has been described in dermatophytes. Altogether, our investigations demonstrate the importance of HSP networks for cell adaptation as another key, intrinsic aspect of the biology of *T. rubrum*, indicating such proteins as potential drug molecular targets for antifungal therapy against dermatophytes.

## Figures and Tables

**Table 1:** Set of primers used in RT and qPCR analyses.

Gene ID	Putative Annotation	Sequence 5′-3′	Concentration (nM)	Size (bp)
TERG_03206 RT-PCR	*hsp7*-*like*	F: ATGCTCGCCTCAAGAATCTG R: ATCAATACCGATGACCTGGC	-	241/153
TERG_03206 qPCR	*hsp7*-*like*	F: CCAGGTCATCGGTATTGATCTT R: CGGATGGAGTGGTTCTTGTT	100	110
TERG_03206 qPCR	*hsp-7 like* intron-1	F: GTAAGTAGCAAGCTGCAAGG R: CTAGACGGTTAAATGTCAGTCAAA	100	88
TERG_01883 qPCR	*hsp75*-*like*	F: GAAAGAGGTTGCCGAGACTAAG R: CTCTGGTTGTCGTTGAAGTAGG	300	84
TERG_01659 qPCR	*hsp30*	F: AAAGTTCGATGTGAGGGAGTG R: GAGTGTTTGAGGATCGGTGAA	100	106
TERG_04141 qPCR	*hsp60*	F: GATTGCCCAGGTTGCTACTAT R: AGTGATGACACCTTCCTTTCC	100	100
TERG_06963 qPCR	*hsp90*	F: GGTCAACAACCTCGGTACTATT R: GACACCGAACTGTCCAATCA	300	100
TERG_07658 qPCR	*hsp70-like*	F: TGAGATGGTCGGTGGATGTA R: GGTTTAGGGTGAAGGAGAGTTG	300	89
TERG_07949 qPCR	*hsp104*	F: TGTCGAGAAGGACGAAGAATAAC R: GGAACATCGCCTCTGACTATC	300	106
TERG_12507 qPCR	*hsp104-like*	F: GCTGATGGATGATGGTCGTATC R: GTTGGGCGGTTCAGGTATT	100	111

## Supplementary Materials

The following are available online at https://www.mdpi.com/2073-4409/8/10/1206/s1, Table S1: Read counts in intron regions of HSP genes after *T. rubrum* exposure to UDA, Figure S1: Full-length gel concerning Figure 2, Figure S2: Schematic representation of Hsp104 paralogs in *T. rubrum*, Figure S3: Schematic representation of TERG_07658, an Hsp70 family member.
