# Supplementary material for: Alternative Splicing in Heat Shock Protein Transcripts as a Mechanism of Cell Adaptation in Trichophyton rubrum
Source: Cells. 2019 Oct 5;8(10):1206. doi: 10.3390/cells8101206 (PMC6830096; doi:10.3390/cells8101206)
Supplement: Supplementary file 1 [file cells-08-01206-s001.pdf]

Table S1. Read counts in intron regions of HSP genes after *T. rubrum* exposure to UDA.

Gene ID	Intron	Putative annotation	0h	3h UDA	12h UDA
**TERG_01883**	Intron-1	hsp75-like protein	3390	0	15
**TERG_03206**	Intron-1	hsp7-like protein	2748	0	42

*Data described by Mendes et al (2018).

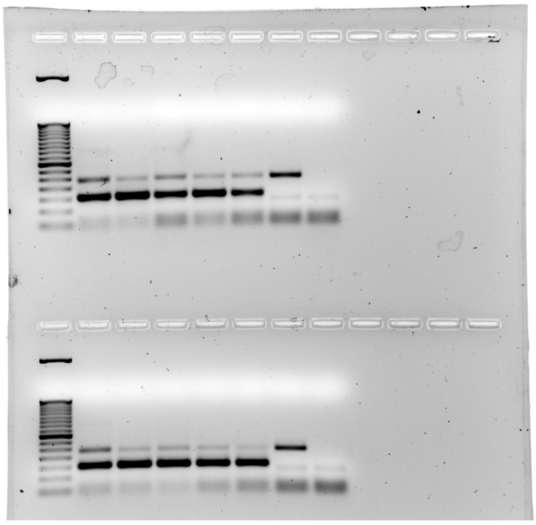

Figure S1. Full-length gel concerning Figure 2.

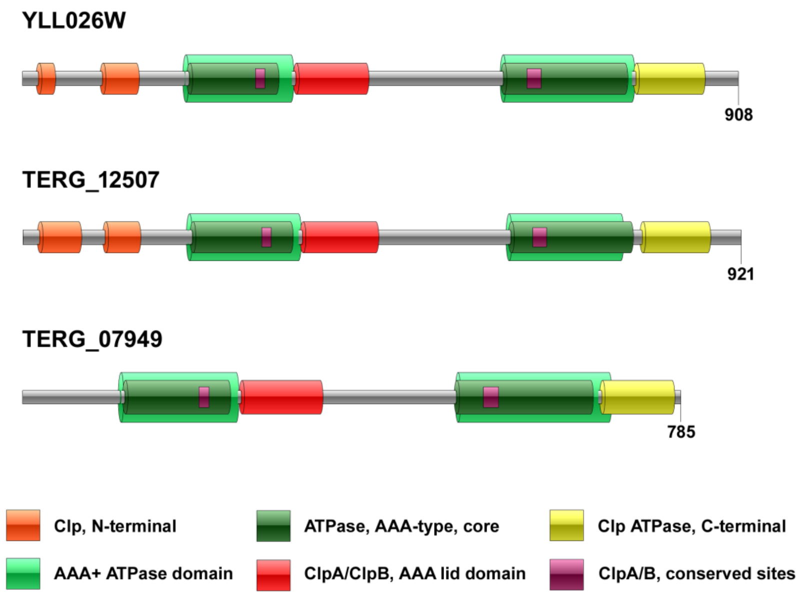

Figure S2. Schematic representation of TERG_07949 and TERG_012507 proteins in comparison to the Hsp104 found in *Saccharomyces cerevisiae* (YLL026W). Conserved sites and domains are indicated in the structure of the proteins. *T. rubrum* sequences can be considered as paralogs, and orthologous to the *Hsp104* gene in *S. cerevisiae*.

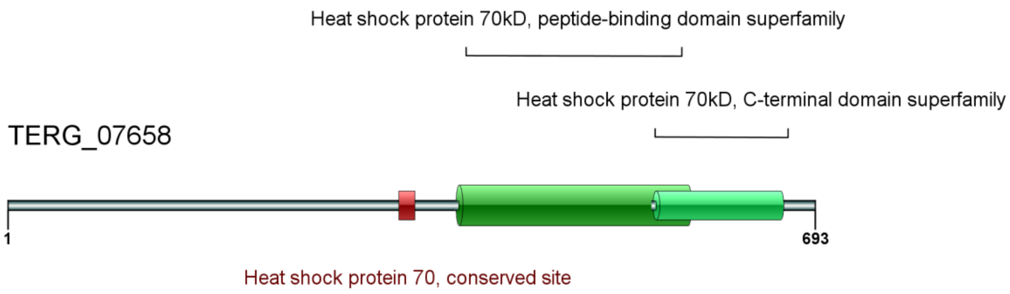

Figure S3. Schematic representation of TERG_07658 protein. *In silico* analyses reveal that despite identified as *hsp88,* this gene actually belongs to the Hsp70 family. Conserved sites and domains are indicated in the structure.
